# Molecular Control of Neural Stem Cell Quiescence in the Adult Zebrafish Telencephalon: Established Mechanisms and an Integrative Hypothesis

**DOI:** 10.1002/glia.70234

**Published:** 2026-10-06

**Authors:** Mathilde Hoareau, Nicolas Diotel, Sepand Rastegar

**Affiliations:** ^1^ Institute of Biological and Chemical Systems‐Biological Information Processing (IBCS‐BIP) Karlsruhe Institute of Technology (KIT) Karlsruhe Germany; ^2^ UMR 1188, Diabète athérothrombose Thérapies Réunion Océan Indien (DéTROi) Université de La Réunion, INSERM Saint‐Pierre France

**Keywords:** adult neurogenesis, neural stem cell, quiescence, radial glial cell, telencephalon, zebrafish

## Abstract

The zebrafish telencephalon is an excellent model for adult neurogenesis under physiological and regenerative conditions, as many pathways regulating neural stem cell behavior are conserved with mammalian neurogenic niches. Resident radial glial cells (RGCs) serve as neural stem cells in the adult brain and are readily accessible in vivo, because forebrain eversion repositions the ventricular zone to the dorsal surface. Recent studies have revealed substantial heterogeneity among RGCs, including activation‐primed cells and deeply quiescent states during homeostasis. Here, we first review the evidence that four mechanisms control quiescence in pallial RGCs: Notch3‐mediated lateral inhibition, the neuronal bone morphogenetic protein (BMP)‐Id1 axis, the nuclear receptor Nr2f1b, and the secreted factor Midkine‐a. The evidence supporting them is unequal, and we state its level for each before asking whether they act together. Notch‐mediated lateral inhibition and neuronal BMP‐Id1 signaling converge on *her4.1*, the clearest documented interaction among them, whereas Nr2f1b establishes a deeper dormancy program that resists Notch and BMP inhibition and protects a reserve stem cell pool. Midkine‐a is proposed to reinforce quiescence, pending functional validation. Finally, we propose as a hypothesis that these mechanisms act as partially redundant inputs whose relative contributions determine quiescence depth and vary along the antero‐posterior axis of the pallium. Together, these findings reframe quiescence as a spectrum of actively maintained conditions rather than a passive default state. These mechanisms are conserved across vertebrates, with implications for understanding why mammalian neural stem cell pools decline with age and injury.

## Graphical abstract

- Adult zebrafish radial glia quiescence is controlled by at least four mechanisms: Notch3, BMP‐Id1, Nr2f1b and Midkine‐a.
- We propose these act as partially redundant inputs, biased along the antero‐posterior axis that set quiescence depth.

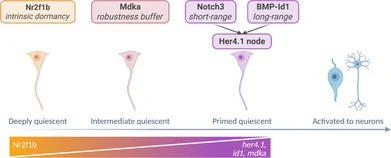

## 1 Introduction

A central unresolved question is how multiple regulatory pathways are integrated at the level of individual cells to produce a continuous spectrum of quiescence depths, from highly responsive, primed states to deeply dormant, activation‐resistant ones. This question is not merely conceptual; it is a prerequisite for designing strategies to reactivate neural stem cells (NSCs) in settings where endogenous regeneration is insufficient, such as the aging or injured mammalian brain. This review addresses that question by examining the four principal regulatory mechanisms identified so far in the adult zebrafish telencephalon. Importantly, we explore how these mechanisms interact to generate a continuum of states, rather than acting as independent parallel switches. Throughout, we distinguish experimentally established findings from the mechanistic hypotheses we propose, presenting the latter as falsifiable predictions intended to guide future experiments rather than as established conclusions. Notably, Section 4 sets out what has been established for each mechanism; Section 5 asks what is known about their interaction; and Section 6 develops, explicitly as a hypothesis, an integrative framework and the predictions that would refute it.

The zebrafish telencephalon has emerged as one of the most tractable systems for addressing this question, combining experimental advantages unique to teleosts with a molecular architecture broadly conserved with mammalian forebrain neurogenic niches. The zebrafish offers external embryonic development, optical transparency, a sophisticated genetic toolkit including the Gal4/UAS system, Cre/loxP lineage tracing, and efficient CRISPR/Cas9 genome editing, as well as deep conservation with human biology, with more than 70% of human genes having at least one zebrafish orthologue (Carney and Mosimann 2018; Daniel et al. 2023; Howe et al. 2013; Kawakami et al. 2016). Crucially, unlike the adult mouse brain where constitutive neurogenesis is largely restricted to the subventricular zone (SVZ) and the subgranular zone (SGZ) of the dentate gyrus (Figure 1), the adult zebrafish brain maintains widespread neurogenic activity across approximately 16 proliferative zones distributed along ventricular surfaces throughout the brain (Caron et al. 2022; Chen et al. 2024; Ganz and Brand 2016; Grandel et al. 2006; Lindsey et al. 2018; März et al. 2010). Among these, the telencephalon is the best‐characterized neurogenic domain and the most amenable to in vivo manipulation (Barbosa et al. 2016; Chapouton et al. 2011; Diotel, Rodriguez Viales, et al. 2015; Foley et al. 2024; Lam et al. 2009; März et al. 2010; Rodriguez Viales et al. 2015). Recent advances in single‐cell multi‐omics have demonstrated that adult NSC populations in the zebrafish telencephalon are not homogeneous but exist across multiple molecularly and functionally distinct states whose transitions are governed by tightly coordinated intrinsic programs and extrinsic niche‐derived signals (Cosacak et al. 2019; Katz et al. 2016; Labusch et al. 2020, 2024; Lange et al. 2020; März et al. 2010; Morizet et al. 2025; Than‐Trong et al. 2020).

**FIGURE 1:**
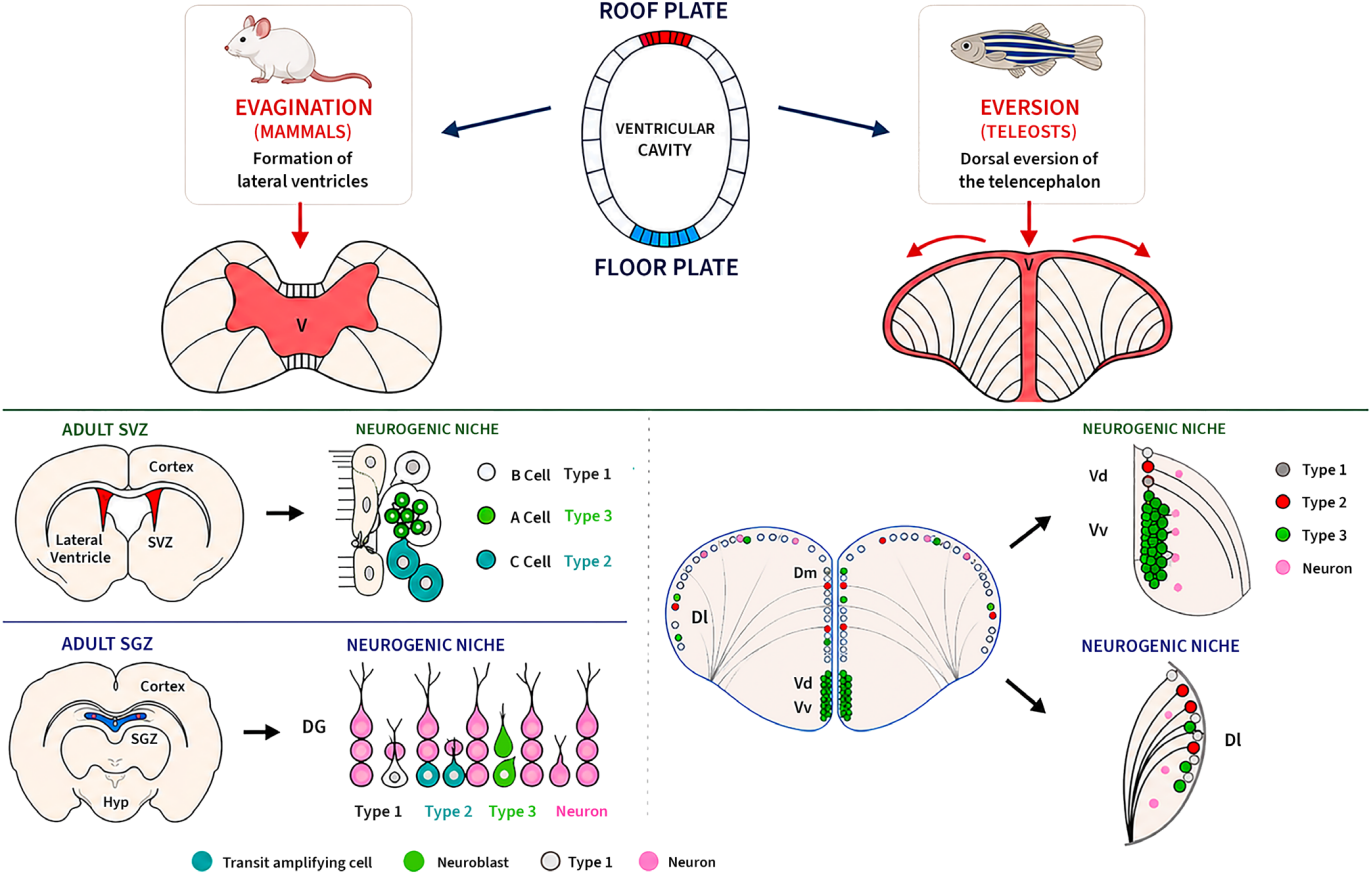
Comparative overview of adult neurogenesis in mammals and teleosts (zebrafish). Top panel: Mammals and teleosts share a conserved neural tube organization in early embryogenesis, with a ventricular cavity bordered by roof and floor plates. Their telencephalic development then diverges: Mammals form paired cerebral hemispheres around the lateral ventricles by evagination, whereas teleosts undergo dorsal eversion, producing an inverted arrangement of pallial subdivisions while retaining homologous developmental territories. Bottom panel: Adult neurogenic niches differ markedly between the two. In mammals, constitutive neurogenesis is largely restricted to two niches: The subventricular zone (SVZ) of the lateral ventricles and the subgranular zone (SGZ) of the dentate gyrus. In both, stem cells progress through a conserved Type 1 → Type 2 → Type 3 → neuron lineage. In zebrafish, neurogenesis remains widespread along the ventricular zone of the adult telencephalon, with stem cells distributed across multiple pallial and subpallial domains—including the dorsal (Vd) and ventral (Vv) subpallium and the lateral pallium (Dl), the latter considered homologous to the mammalian hippocampus. Thus, while adult neurogenesis follows a conserved progression from quiescent stem cells to differentiated neurons across vertebrates, the anatomical distribution, abundance, and regenerative capacity of neurogenic niches differ substantially between mammals and teleosts.

Quiescence, the state of reversible cell‐cycle arrest adopted by most adult stem cells, is not a passive default condition but an actively maintained program that must be continuously reinforced against proliferative stimuli while remaining responsive to physiological demand. In the adult brain, the balance between quiescence and activation determines the size, longevity, and regenerative capacity of NSC pools. Its dysregulation underlies age‐related neurogenic decline and limits repair after injury. Understanding how quiescence is molecularly established and maintained is therefore a central question in adult stem cell biology with direct therapeutic relevance.

## 2 Anatomical Organization of the Adult Zebrafish Telencephalon

The adult zebrafish telencephalon is structurally and functionally subdivided into two major domains: the dorsal telencephalon (pallium) and the ventral telencephalon (subpallium). These regions differ in developmental origin, cellular composition, and neurogenic dynamics, reflecting distinct organizational principles within the forebrain (Ganz et al. 2012; Hegarty et al. 2024; Yanez et al. 2022).

### 2.1 Evolutionary Eversion of the Forebrain

A defining feature of teleost brain development is forebrain eversion, which contrasts fundamentally with the evagination‐based morphogenesis observed in mammals (Folgueira et al. 2012; Folgueira and Clarke 2024; Yanez et al. 2022) (Figure 1). In zebrafish embryos, the lateral walls of the neural tube expand dorsolaterally around the midline, producing an outward folding of the telencephalic tissue rather than the formation of internal hemispheric cavities. As a result, the ventricular zone is relocated from an internal position to an extensive epithelial surface along the dorsal aspect of the telencephalon, while the ventricular cavity is correspondingly reduced and partially covered by the thin, non‐neurogenic tela choroidea (Folgueira et al. 2012). In contrast, mammalian forebrain development proceeds through evagination, in which the telencephalic walls balloon outward to form enclosed hemispheres lined internally by the lateral ventricular system (Figure 1). This fundamental topological difference means that neural stem cells in the mammalian SVZ line an internal ventricular surface embedded within the brain parenchyma, whereas zebrafish radial glial cells (RGCs) sit at or near the outer brain surface, a configuration that is uniquely accessible for in vivo imaging, targeted genetic manipulation, and longitudinal lineage‐tracing studies (Cosacak et al. 2019; Dray et al. 2015; Katz et al. 2016; Labusch et al. 2020, 2024; Lange et al. 2020; Morizet et al. 2025; Than‐Trong et al. 2020).

### 2.2 The Dorsal Telencephalon

The dorsal telencephalon shares broad developmental, molecular, and functional similarities with the cortical and hippocampal regions of the mammalian brain (Ganz et al. 2014; Hegarty et al. 2024; Hiraki‐Kajiyama et al. 2024; Yanez et al. 2022). It is primarily composed of excitatory glutamatergic neurons and contains one of the best‐characterized adult neurogenic niches among vertebrates (Adolf et al. 2006; Chapouton et al. 2011; Foley et al. 2024; Ganz and Brand 2016; Grandel et al. 2006). The ventricular zone lining the dorsal surface is populated predominantly by RGCs, which serve as the principal neural stem cell population in the adult zebrafish brain (Figure 1) (Chapouton et al. 2007, 2010, 2011; Diotel et al. 2016; Edelmann et al. 2013; März et al. 2010; Pellegrini et al. 2007). Although adult pallial stem cells originate from distinct embryonic progenitor populations (Dirian et al. 2014), they exhibit a characteristic bipolar morphology in which the soma resides at the ventricular surface. From this apical position, a short process extends into the cerebrospinal fluid, while a long basal process spans the entire depth of the pallial tissue and anchors to the pial surface (Grupp et al. 2010; Jurisch‐Yaksi et al. 2020; Lindsey et al. 2018; März et al. 2010; Pellegrini et al. 2007). Interestingly, the short process terminates in an apical primary cilium whose configuration is heterogeneous, with monociliated and multiciliated RGCs coexisting at the ventricular surface (D'Gama et al. 2021). The dorsolateral and dorsoposterior regions of the zebrafish telencephalon have been proposed as homologues of the mammalian hippocampus, the latter containing Type 1 (radial glia‐like), Type 2 (transient amplifying), and Type 3 (neuroblast) progenitor cells that give rise to neurons (Figure 1).

In zebrafish, under homeostatic conditions, the majority of dorsal RGCs remain quiescent, dividing only infrequently to preserve the long‐term integrity of the stem cell pool (Adolf et al. 2006; Chapouton et al. 2010; Edelmann et al. 2013; Kaslin et al. 2009; Labusch et al. 2020; Morizet et al. 2025; Rodriguez Viales et al. 2015). This quiescent population is not uniform, and the molecular basis of its heterogeneity is the central subject of this review.

### 2.3 The Ventral Telencephalon

The ventral telencephalon is more closely related to basal forebrain structures in mammals, including regions analogous to the striatum and septum (Mueller et al. 2008; Wullimann and Rink 2002). In contrast to the pallium, it is dominated by inhibitory GABAergic neuronal populations and exhibits a distinct pattern of neurogenic organization (Hegarty et al. 2024; Mueller et al. 2008; Wullimann and Rink 2002). Neural progenitor cells in the subpallium differ markedly from RGCs in both morphology and proliferative behavior (Figure 1). Rather than displaying elongated radial processes, these cells retain a more epithelial‐like, compact and cuboidal architecture with limited extension into the surrounding parenchyma, and they exhibit substantially higher baseline proliferative activity compared with their pallial counterparts (Adolf et al. 2006; de Oliveira‐Carlos et al. 2013; Ganz et al. 2010; Lindsey et al. 2018; März et al. 2010). Consequently, the subpallial niche is constitutively active, continuously generating neuronal precursors that contribute to local forebrain and olfactory circuitry. This region displays similarities to the mammalian SVZ that contains B cells (Type 1 neural stem cells), C cells (Type 2 transient amplifying progenitors), and A cells (Type 3 neuroblasts), which give rise to new neurons in mammals (Figure 1). The mechanisms regulating subpallial progenitor proliferation are beyond the scope of this review, which focuses on the quiescence regulation of dorsal pallial RGCs.

## 3 Cellular Properties, Division Modes, and Heterogeneity of Dorsal RGCs

Adult zebrafish RGCs in the dorsal telencephalon represent the functional and molecular homologues of adult neural stem cells in the mammalian ventricular‐SVZ, particularly B1 astrocytes in the mouse brain (Chen et al. 2024; Diotel et al. 2020; Ghaddar et al. 2021). In contrast to mammals, the teleost brain lacks typical stellate parenchymal astrocytes; ventricular RGCs therefore fulfill both stem cell and homeostatic glial functions (Diotel et al. 2020; Jurisch‐Yaksi et al. 2020; Morizet et al. 2024). This dual identity is reflected in a molecular signature that integrates three functional modules: neural stem cell competence, structural support, and metabolic homeostasis. Stem cell identity is conferred by transcription factors Sox2 and the cytoskeletal proteins Nestin and Vimentin, which maintain proliferative potential and radial morphology (März et al. 2010). Structural integrity of the elongated bipolar process is provided by Gfap, while Blbp (Fabp7) supports lipid transport along this process and additionally marks a subset of quiescent cells (Diotel et al. 2016; Lam et al. 2009; März et al. 2010; Morizet et al. 2025). Metabolic and homeostatic support functions, characteristic of periventricular astrocytes, are encoded by (i) Glast (Slc1a3), which clears extracellular glutamate to prevent excitotoxicity, (ii) Glutamine synthetase (GS), which recycles glutamate into glutamine, (iii) S100*β* and Aqp4, which regulate calcium signaling and water homeostasis respectively, and Cyp19a1b (aromatase B), which marks the neurogenic ventricular zone and confers estrogen production and sensitivity (Diotel, Le Page, et al. 2010; Diotel, Vaillant, et al. 2010; Diotel et al. 2016; Edelmann et al. 2013; Gleiser et al. 2016; Jurisch‐Yaksi et al. 2020; März et al. 2010; Pellegrini et al. 2007; Tong et al. 2009). The co‐expression of all three modules within a single cell positions RGCs as uniquely suited to integrate niche signals, maintain tissue homeostasis, and retain long‐term regenerative capacity simultaneously (Figure 2, Table 1).

**FIGURE 2:**
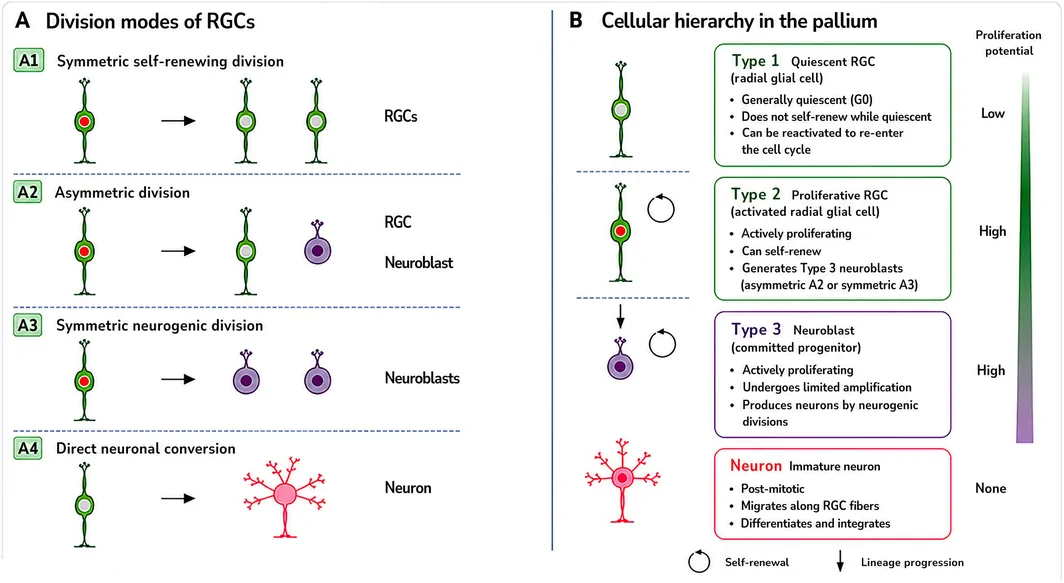
Cellular hierarchy, division modes, and molecular identity of radial glial cells during adult neurogenesis in the zebrafish pallium. (A) Radial glial cells (RGCs) exhibit four distinct “division” modes. They can undergo symmetric self‐renewing divisions to expand the stem cell pool (A1), asymmetric divisions generating one radial glial cell and one neuroblast (A2), symmetric neurogenic divisions producing two neuroblasts (A3), or direct neuronal conversion without cell division (A4). (B) Classical cellular hierarchy of the adult zebrafish pallium. Quiescent radial glial cells (Type 1) remain in a non‐cycling state but can be reactivated to enter the cell cycle. Activated proliferative radial glial cells (Type 2) self‐renew while generating committed neuroblasts (Type 3a/b), which undergo transit amplification before differentiating into post‐mitotic neurons. Self‐renewal capacity decreases progressively along the lineage, whereas neuronal differentiation increases.

**TABLE 1:** Molecular markers of neural stem cell lineage states in the adult zebrafish telencephalon.

Cell type	Proliferative state	Structural/glial markers	Stem cell/proneural markers	Quiescence markers	References
Type 1 quiescent RGC	PCNA− BrdU label‐retaining (slowly cycling)	*gfap*+, Vimentin+, *blbp*+, *glast*+, GS+, S100*β*+, Aqp4+, ZO‐1+, *cyp19a1b*+, *cxcr4*+	*sox2*+, Nestin+, *notch3*+	*her4.1*+, *hey1*+, *id1*+, *mdka*+, *nr2f1b*+ (deeply quiescent subset only)	März et al. 2010; Pellegrini et al. 2007; Chapouton et al. 2010, 2011; Diotel, Le Page, et al. 2010; Diotel et al. 2016; Rodriguez Viales et al. 2015; Zhang et al. 2020, 2021; Lübke et al. 2022; Morizet et al. 2025; Chen et al. 2026
Type 2 Activated RGC	PCNA+, BrdU+ (actively cycling)	*gfap*+, Vimentin+, *blbp*+, *glast*+, GS+, S100*β*+, Aqp4+, ZO‐1+, *cyp19a1b*+, *cxcr4*+	*sox2*+, Nestin+, *ascl1a* + (proneural), *deltaA*+ (fate‐decision subset)	*her4.1* low/−, *id1* low/−	März et al. 2010; Pellegrini et al. 2007; Chapouton et al. 2011; Diotel, Le Page, et al. 2010; Diotel et al. 2016; Rodriguez Viales et al. 2015; Zhang et al. 2020, 2021; Barbosa et al. 2015; Than‐Trong et al. 2020; Mancini et al. 2023
Type 3a Neuroblast	PCNA+	*gfap* low, *blbp* low, *cyp19a1b* low	*sox2*+, Nestin+, PSA‐NCAM+	(–)	März et al. 2010
Type 3b Neuroblast	PCNA+	(–)	*sox2*+, PSA‐NCAM+	(–)	März et al. 2010
Neuron	PCNA−	HuC/D+, MAP2a + b+	(–)	(–)	Kroehne et al. 2011; Pellegrini et al. 2007

*Note:* +, expressed/detected; −, not detected; low, reduced expression; −, not applicable.

Abbreviation: RGC, radial glial cell.

Within this framework, RGCs and neural progenitors do not represent a uniform population but exist across a spectrum of molecular states associated with distinct proliferative behaviors (Figure 2). During homeostasis, the majority of dorsal RGCs reside in a quiescent state where they maintain niche architecture while remaining largely non‐proliferative (Alunni et al. 2013; Chapouton et al. 2010, 2011; de Oliveira‐Carlos et al. 2013; Edelmann et al. 2013; Labusch et al. 2024; Zhang et al. 2020, 2021). Upon activation, RGCs most commonly undergo asymmetric division, producing one self‐renewing stem cell and one intermediate amplifying progenitor (IAP) marked by Ascl1a, which undergoes a limited number of proliferative cycles before generating migratory neuroblasts. Symmetric divisions also occur, either expanding the stem cell pool or producing committed progenitors, depending on local niche signals and spindle orientation (Rothenaigner et al. 2011; Than‐Trong and Bally‐Cuif 2015; Than‐Trong et al. 2020). Evidence further suggests that a subset of RGCs may undergo direct neuronal conversion without an intermediate proliferative stage (Barbosa et al. 2015; Rosa et al. 2024; Rothenaigner et al. 2011) (Figure 2).

Lineage analyses and immunostainings have demonstrated that the dorsal ventricular zone is organized into a stepwise cellular hierarchy comprising three principal functional states (Lange et al. 2020; März et al. 2010; Rothenaigner et al. 2011; Than‐Trong et al. 2020). Quiescent radial glia (Type 1) are slowly cycling stem cells expressing Gfap, Blbp, Glast, GS, zonula occludens‐1 (ZO‐1), and Sox2, while lacking the proliferation marker proliferating cell nuclear antigen (PCNA). Upon activation, they transition into proliferative progenitors (Type 2), which retain glial identity but re‐enter the cell cycle. These cells give rise to neuroblasts (Type 3), which express polysialylated neural cell adhesion molecule (PSA‐NCAM) and complete final mitotic divisions prior to differentiation into mature neurons (Chen et al. 2024; Diotel et al. 2020; März et al. 2010) (Table 1).

Single‐cell transcriptomic studies have refined this view by demonstrating that quiescence itself exists along a continuum of molecular states rather than as a single uniform condition (Cosacak et al. 2019; Kutsia et al. 2022; Labusch et al. 2024; Lange et al. 2020; Morizet et al. 2025; Than‐Trong and Bally‐Cuif 2015; Than‐Trong et al. 2020) (Figure 3, Table 2). At one end are primed quiescent RGCs: transcriptionally active and highly responsive to niche‐derived cues, expressing *her4.1*, *id1*, and *mdka* and poised for rapid reactivation (Alunni et al. 2013; Foley et al. 2024; Lange et al. 2020; Lübke et al. 2022; Rodriguez Viales et al. 2015; Than‐Trong et al. 2018; Zhang et al. 2021). At the opposite end are deeply quiescent RGCs: reduced transcriptional activity, elevated activation thresholds, limited responsiveness to niche fluctuations, and enrichment for Nr2f1b together with other dormancy‐associated molecular signatures (Foley et al. 2024; Labusch et al. 2020; Morizet et al. 2025; Than‐Trong et al. 2018). A critical insight from recent studies is that cells at different positions along this spectrum respond qualitatively differently to the same niche perturbations, a finding that demands a mechanistic explanation going beyond the simple enumeration of individual pathways. Understanding this integration is the central subject of the following section.

**FIGURE 3:**
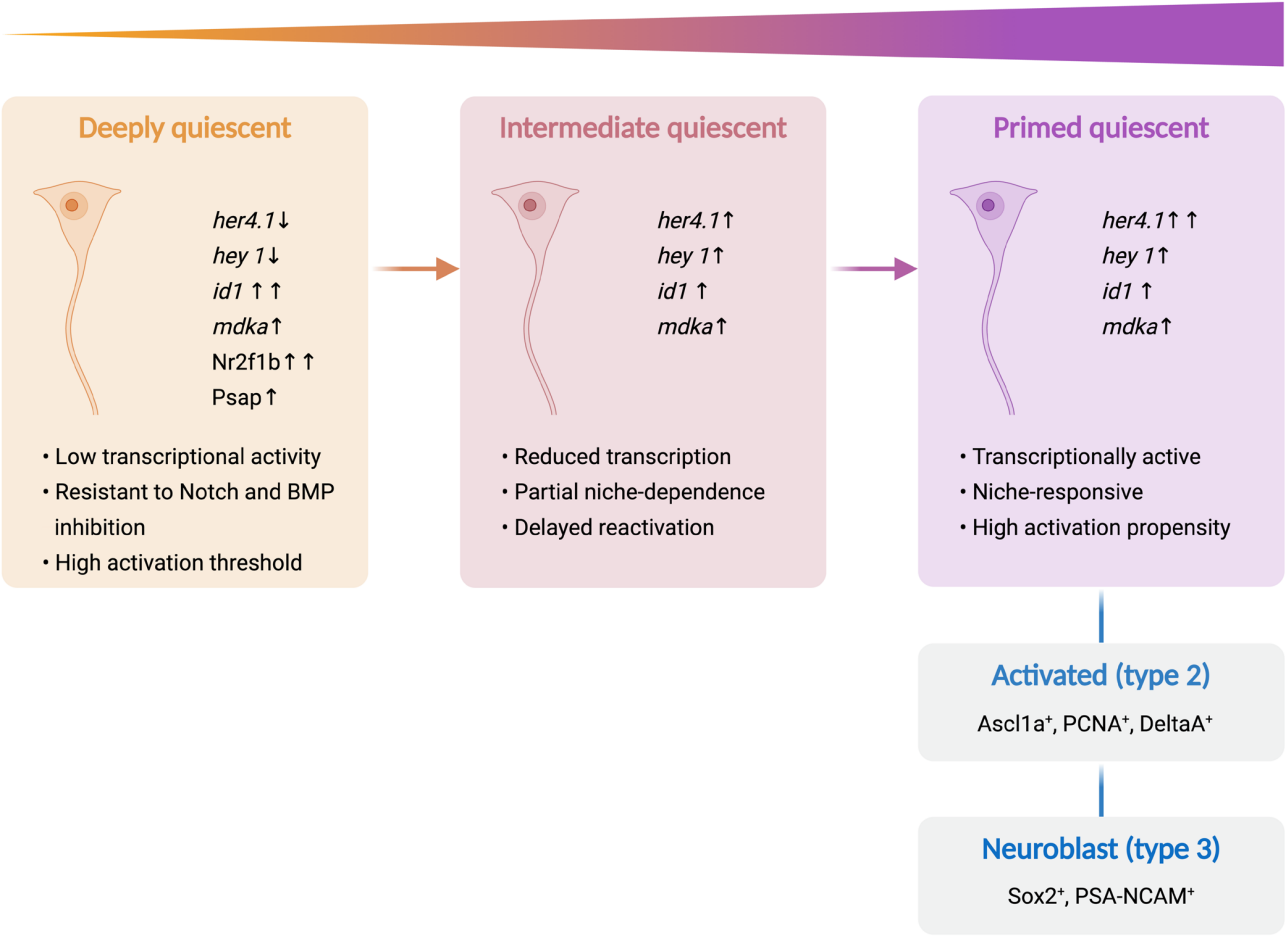
The quiescence continuum is depicted as three RGC states spanning a gradient from deeply quiescent to primed quiescent cells. Expression of the principal molecular markers (*her4.1*, *id1*, *mdka*, and *nr2f1b*) shifts progressively along this continuum. Upon activation, primed quiescent RGCs transition toward Type 2 and Type 3 neuroblasts. Note that ↓ indicates reduced expression, ↑ increased expression, and ↑↑ strongly increased expression. Created in BioRender. Hoareau (2026) https://BioRender.com/8qc62po.

**TABLE 2:** Molecular signatures along the RGC quiescence spectrum in the adult zebrafish telencephalon.

Quiescence state	Notch pathway markers	BMP pathway markers	Cell‐intrinsic markers	Functional features	References
Primed quiescent	*her4.1* high *hey1*+ *notch3*+	*id1* moderate pSmad1/5 moderate	*ascl1a* low/detectable *nr2f1b*−	High responsiveness to niche signals; rapid cell‐cycle re‐entry; sensitive to Notch/BMP inhibition; highest activation propensity	Chapouton et al. 2010; Than‐Trong et al. 2018, 2020; Morizet et al. 2025
Intermediate quiescent	*her4.1* high *hey1*+ *notch3*+	*id1* moderate–high pSmad1/5 moderate	*ascl1a*− *nr2f1b* −/low	Delayed response to activation signals; Notch3–Her4.1 + BMP–Id1 co‐engaged; Her4.1 convergence zone	Zhang et al. 2021; Cosacak et al. 2019; Morizet et al. 2025
Deeply quiescent	*her4.1* low (Notch‐independent) *hey1* low/−	*id1* high pSmad1/5 low	*nr2f1b*++ Prosaposin+ *ascl1a*−	Resistant to Notch + BMP inhibition; cell‐intrinsic lock via Nr2f1b; long‐term reserve pool; requires strong perturbation to re‐enter cell cycle	Morizet et al. 2025; Labusch et al. 2024

*Note:* High/moderate/low, relative expression level; +, expressed; −, not detected; ++, strongly expressed.

Abbreviation: RGC, radial glial cell.

## 4 Molecular Mechanisms Controlling RGC Quiescence: The Experimental Evidence

The existence of a quiescence continuum implies that no single pathway explains RGC behavior across all states. At least four molecular mechanisms have so far been implicated in the control of quiescence in pallial RGCs: Notch3‐mediated lateral inhibition, the neuronal bone morphogenetic protein (BMP)‐Id1 axis, the cell‐intrinsic nuclear receptor Nr2f1b, and the secreted factor Midkine‐a (Table 3). They are not equally well supported. Notch3 and BMP‐Id1 rest on convergent loss‐ and gain‐of‐function data, Nr2f1b on a single but functionally direct study, and Midkine‐a on expression and *cis*‐regulatory analysis alone. We describe each in turn and state what has been demonstrated and what has not. Their interaction is treated in Section 5, and the framework we propose in Section 6. Other signals also influence proliferation in the pallial germinal zone, including Fgf (Ganz et al. 2010), Cxcl12/Cxcr4 (Diotel, Vaillant, et al. 2010), estrogen (Pellegrini et al. 2007; Tong et al. 2009; Diotel et al. 2013), insulin signaling (Gence et al. 2023), and injury‐induced inflammatory signals (Kyritsis et al. 2012). We focus on the four mechanisms above because they are the ones for which quiescence itself, rather than proliferation in general, has been addressed by both expression and perturbation experiments.

**TABLE 3:** Molecular mechanisms implicated in the control of radial glial cell quiescence in the adult zebrafish pallium.

Mechanism	Signal source and spatial range	Molecular readout	Perturbation that operationally distinguishes it	Principal experimental support	Evidence status and open questions
1. Notch3 lateral inhibition	Activated progenitors (Jag1b, DeltaA); short‐range, requires cell contact	*her4.1*, *hey1*	Output lost upon Notch inhibition; effect confined to RGCs contacting activated progenitors	Pharmacological and genetic Notch inhibition increases RGC proliferation; *her4.1* and *hey1* act downstream (Alunni et al. 2013; Chapouton et al. 2010; Than‐Trong et al. 2018)	Established by loss and gain of function. Identity and dynamics of the operative ligands not resolved at single‐cell level
2. BMP‐Id1 axis	Differentiated neurons (BMPs); long‐range, diffusible, tissue‐wide	*id1* (via Smad1/5/4)	Output lost upon BMP inhibition (DMH1), which also lowers *her4.1*	BMP overexpression raises *id1*; DMH1 lowers *id1* and increases proliferation; a conserved BMP‐responsive element drives *id1* in the telencephalon (Zhang et al. 2020, 2021)	Established by loss and gain of function. Direct action of neuron‐derived BMP on RGCs not demonstrated in vivo
3. Nr2f1b deep dormancy	Cell‐intrinsic; population enriched caudally	Nr2f1b; *fabp7*	State persists after Notch inhibition, and after combined Notch and BMP inhibition, under the conditions tested	The most deeply quiescent cluster resists Notch blockade; Nr2f1b gain of function maintains, and reduction of its activity releases, this resistance (Morizet et al. 2025)	Established for the perturbations tested. Direct transcriptional targets unknown; reserve function not tested by lineage tracing
4. Midkine‐a (candidate)	Quiescent RGCs (secreted); local, paracrine or autocrine	*mdka*	Expression insensitive to inhibition of Notch or BMP individually	Expressed in quiescent RGCs in a pattern overlapping *id1*, in constitutive and reactive neurogenesis; multiple *cis*‐regulatory elements integrate several transcription factors (Lübke et al. 2022; Chen et al. 2026)	Candidate. Expression and *cis*‐regulation established; loss of function not performed; receptor and effector pathway unknown

### 4.1 Notch3 Signaling: Short‐Range Control by Neighboring Activated Progenitors

The best‐characterized of the four mechanisms is Notch signaling, which establishes a local population‐sensing mechanism through direct cell‐to‐cell contact (Alunni et al. 2013; Chapouton et al. 2010; Than‐Trong et al. 2018). The principal source of Notch ligands is neighboring activated progenitors. Through dynamic interactions with these cells, Notch3 signaling in adjacent RGCs activates the downstream effectors *her4.1* and *hey1*, basic helix–loop–helix (bHLH) transcriptional repressors that arrest cell‐cycle progression and preserve stem cell identity (Diotel et al. 2020; Foley et al. 2024; Kroehne et al. 2011; Ortica et al. 2026; Takke et al. 1999; Than‐Trong et al. 2018). Jagged1b and DeltaA have been identified as candidate ligands whose spatiotemporal expression patterns contribute to this regulation (Foley et al. 2024; Mancini et al. 2023; Ortica et al. 2026; Than‐Trong et al. 2018).

This circuit functions as lateral inhibition. Indeed, by allowing newly activated progenitors to transiently suppress the activation of adjacent RGCs, it prevents simultaneous recruitment of neighboring stem cells and protects the ventricular zone from rapid clonal exhaustion. However, the range of this signal is inherently limited to cells in direct contact with activated progenitors. Notch signaling alone therefore cannot account for the quiescence of cells that are distant from or not in contact with activated progenitors. This spatial limitation is one reason why longer‐range mechanisms have been sought.

### 4.2 The Neuronal BMP‐Id1 Axis: Long‐Range Control by Differentiated Neurons

A second, independently established mechanism is provided by BMPs secreted by differentiated neurons distributed throughout the pallial parenchyma (Zhang et al. 2021). These diffusible signals reach RGCs at the ventricular surface, where they activate canonical BMP signaling and drive expression of *id1* through conserved Smad1/5/4‐responsive elements in the *id1* upstream regulatory region (Diotel, Beil, et al. 2015; Diotel et al. 2020; Rodriguez Viales et al. 2015; Zhang et al. 2020, 2021). Id1, a dominant‐negative helix–loop–helix (HLH) protein that lacks a DNA‐binding domain, inhibits pro‐proliferative bHLH transcription factors by sequestering them and preventing their interaction with target gene promoters, thereby establishing a transcriptional barrier to cell‐cycle entry (Rodriguez Viales et al. 2015). Through this mechanism, differentiated neurons establish a tissue‐wide inhibitory signal that couples the size of the neuronal pool to the quiescence level of the stem cell compartment, long‐range negative feedback that buffers the entire niche against excessive activation. BMP overexpression increases *id1* levels in RGCs, whereas inhibition of BMP signaling with the BMP Type I receptor inhibitor DMH1 reduces *id1* expression and is accompanied by increased proliferation, confirming that the neuron‐to‐RGC BMP‐Id1 axis is functionally active under homeostatic conditions (Zhang et al. 2020, 2021).

### 4.3 Nr2f1b: A Cell‐Intrinsic Program in Deeply Quiescent, Caudally Enriched RGCs

A third mechanism was identified by challenging the quiescent clusters of the adult pallium with pharmacological inhibition of Notch signaling and following their response by single‐cell transcriptomics (Morizet et al. 2025). Most clusters responded, but one cluster, predicted from its transcriptome to be the most deeply quiescent, resisted activation. This cluster is enriched for the orphan nuclear receptor Nr2f1b, and functional experiments established a causal role: gain of Nr2f1b function prevents activation after Notch blockade, whereas reducing its activity renders these cells responsive. These RGCs are also enriched caudally, along a gradient opposite to that of proliferation, which is highest rostrally (Morizet et al. 2025), so deep quiescence in the pallium has a spatial as well as a molecular dimension. We return to this in Section 6.2.

Two limitations bound what can be concluded. First, the perturbations tested were inhibition of Notch signaling and, in combination, inhibition of BMP signaling with DMH1, which produced no further increase in activation. It does not follow that this state is independent of extrinsic signals in general. The authors of the primary study note that the concentration of gamma‐secretase inhibitor used may already have produced a maximal effect, so that an additive contribution of BMP inhibition could not have been detected under those conditions (Morizet et al. 2025). A contribution of BMP signaling, or of the many extrinsic inputs that were not manipulated, therefore cannot be excluded, and we do not describe this mechanism as extrinsic‐signal‐independent. Second, the direct transcriptional targets of Nr2f1b in zebrafish RGCs have not been characterized.

This population is generally interpreted as a long‐term reserve that protects the stem cell pool over extended timescales. That interpretation has not itself been tested: whether these cells are preferentially recruited once the primed compartment is depleted, and whether they contribute disproportionately to long‐term clonal output, would require lineage tracing. Whether cells transition from extrinsically maintained to Nr2f1b‐dependent quiescence as a function of age or activation history is likewise open.

### 4.4 Midkine‐a: A Candidate Fourth Mechanism

A fourth mechanism has been proposed based on the expression and regulation of the secreted heparin‐binding growth factor Midkine‐a (Mdka). Unlike the three mechanisms above, it has not been tested by loss of function, and it is therefore presented here as a candidate. Under homeostatic conditions, *mdka* is expressed in quiescent RGCs of the ventricular zone, displaying a spatial expression pattern overlapping with Id1 during constitutive neurogenesis and also during the reactive neurogenesis that follows injury (Chen et al. 2026; Lübke et al. 2022). Unlike *id1*, *mdka* expression is largely insensitive to inhibition of either BMP or Notch signaling individually (Lübke et al. 2022; Lübke 2022), suggesting that *mdka* transcription is sustained through the convergent activity of multiple signaling inputs such that disrupting any single pathway is insufficient to collapse it. Consistent with this interpretation, analysis of the *mdka* regulatory landscape has revealed multiple *cis*‐regulatory elements that integrate inputs from several transcription factors (Chen et al. 2026). It should be noted that while these expression and regulatory data establish *mdka* as a strong candidate quiescence‐maintenance factor, the direct functional consequences of *mdka* loss, specifically whether *mdka* depletion causes premature RGC activation, require further experimental validation to definitively establish its role in the quiescence program. Should such loss‐of‐function studies confirm this role, *mdka* would represent a constitutive pro‐quiescence signal that persists even when individual niche pathways are perturbed. The established and open aspects of this mechanism are summarized in Table 3. We note in addition that the insensitivity of *mdka* expression to individual inhibition of Notch or BMP is a statement about how *mdka* is regulated, not about how Mdka protein acts. Its receptor in this tissue is unidentified, and nothing follows from it about whether Mdka signals independently of the other three mechanisms.

### 4.5 Summary of the Experimental Evidence

Table 3 summarizes the four mechanisms: for each, the source and range of the signal, the molecular readout, the perturbation that operationally distinguishes it, the principal experimental support, and what remains untested. We place it here, before any discussion of interaction, so that the difference in the level of evidence is explicit.

## 5 Interaction Between the Mechanisms: Evidence and Possible Routes

That four mechanisms each influence quiescence does not establish that they form a system. This section separates three situations: interactions supported by direct evidence, interactions that can be proposed but not demonstrated, and interactions about which nothing is known. Of the six possible pairs among the four mechanisms, one is supported by direct evidence, one is constrained by a negative result, and four are untested.

### 5.1 Direct Evidence: Notch and BMP Converge on *her4.1*

The most direct evidence that any two of the four mechanisms are functionally connected concerns Notch and BMP.

A key conceptual advance in understanding how the Notch and BMP mechanisms interact comes from the observation that inhibition of BMP signaling reduces expression of *her4.1*, a canonical Notch target (Zhang et al. 2021). This unexpected finding indicates that the two pathways do not simply act in parallel on independent downstream targets: BMP signaling contributes, directly or indirectly, to maintaining the output of the Notch pathway, with Her4.1 serving as a shared integration point.

### 5.2 Two Possible Routes to Convergence: Direct and Indirect

Two classes of explanation can account for this observation, and they are not mutually exclusive. In a direct route, a component of the BMP pathway could act directly on either the *her4.1* locus or on the Her4.1 protein. In this scenario, BMP signaling would be required to sustain *her4.1* expression independently of Notch receptor activation. In an indirect route, BMP instead alters the availability of Notch signaling. The ligands are supplied by activated progenitors, so any manipulation that changes their number or state will change *her4.1* as a secondary consequence. The two routes make different predictions and can be distinguished experimentally. The model developed below concerns the direct route only, and the indirect route has not been excluded.

For the direct route, we propose the following mechanism. Like other Hes and Her family members, *her4.1* represses its own transcription by binding N‐box elements in its own promoter, a negative autoregulatory loop that tends to drive *her4.1* expression toward oscillation or extinction in the absence of sustained input (Hirata et al. 2002; Takebayashi et al. 1994). Id1, by forming dominant‐negative complexes with Her4.1, Her4.5, Her6, and Her9, as demonstrated biochemically in zebrafish (Rodriguez Viales et al. 2015), may directly interfere with this *her4.1* autorepression. By preventing Her4.1 from binding its own promoter, BMP/Id1 signaling would allow sustained Notch‐driven *her4.1* expression to be maintained at high levels, stabilizing the quiescent state. This mechanism is analogous to that proposed for Id proteins and Hes1 in the mouse, where Id1 has been shown to attenuate Hes1 autorepression and thereby extend the duration of Hes1‐mediated transcriptional repression (Bai et al. 2007; Boareto et al. 2017; Sueda et al. 2019; Sueda and Kageyama 2020). We stress that this model remains to be validated directly in vivo in the zebrafish system and should be considered a working hypothesis to guide future experimental design.

Under this proposed model, cells receiving both Notch and BMP signals would maintain high and stable Her4.1 levels and exhibit a strongly quiescent, primed phenotype. Conversely, in cells where BMP input is reduced, for example in regions of the ventricular zone distal from differentiated neurons, *her4.1* autorepression would progressively dampen *her4.1* expression, lowering the quiescence threshold and facilitating transitions toward activation. This spatial variation in BMP signal availability could thus contribute to the heterogeneous activation propensities observed across the ventricular zone, generating a mosaic of quiescence states even within a field of cells receiving uniform Notch input: a testable prediction of the model.

### 5.3 Interactions for Which Evidence Is Currently Lacking

For the remaining relationships the evidence is thin or absent. Nr2f1b‐enriched cells continue to express *id1* (Morizet et al. 2025), so the BMP‐Id1 and Nr2f1b mechanisms are co‐active in the same cells, but whether they interact functionally or only coexist is unknown. The epistasis experiment that would resolve it, manipulating BMP signaling within the Nr2f1b‐high population, has not been reported. For Midkine‐a the only relevant observation is negative: *mdka* expression is not abolished by inhibition of Notch or of BMP individually (Lübke et al. 2022; Lübke 2022). As noted in Section 4.4, this constrains how *mdka* is regulated but is silent on how Mdka protein acts. No interaction involving Midkine‐a has been tested directly.

## 6 An Integrative Hypothesis: Quiescence Depth as a Combined and Regionally Biased Output

Everything that follows in this section is a hypothesis. We present it after the evidence, and separately, so that it cannot be mistaken for an established account. Its purpose is to make a refutable proposal that organizes the observations above and identifies the experiments that would discriminate between it and the alternatives.

### 6.1 How the Four Inputs Might Combine Within a Single Cell

We propose that the four mechanisms can be read as a set of control inputs that together deepen quiescence (Figure 4). The ordering implied below is part of the hypothesis and is not itself demonstrated. (i) Notch3 provides local control sensitive to the immediate cellular environment; (ii) BMP‐Id1 extends this to the tissue scale and, through its proposed convergence on Her4.1, stabilizes the output of Notch; (iii) Nr2f1b establishes a cell‐intrinsic program in cells that resist activation after Notch and BMP inhibition; and (iv) *mdka* is proposed to add redundancy, an interpretation that rests on expression and *cis*‐regulatory data and awaits loss‐of‐function analysis.

**FIGURE 4:**
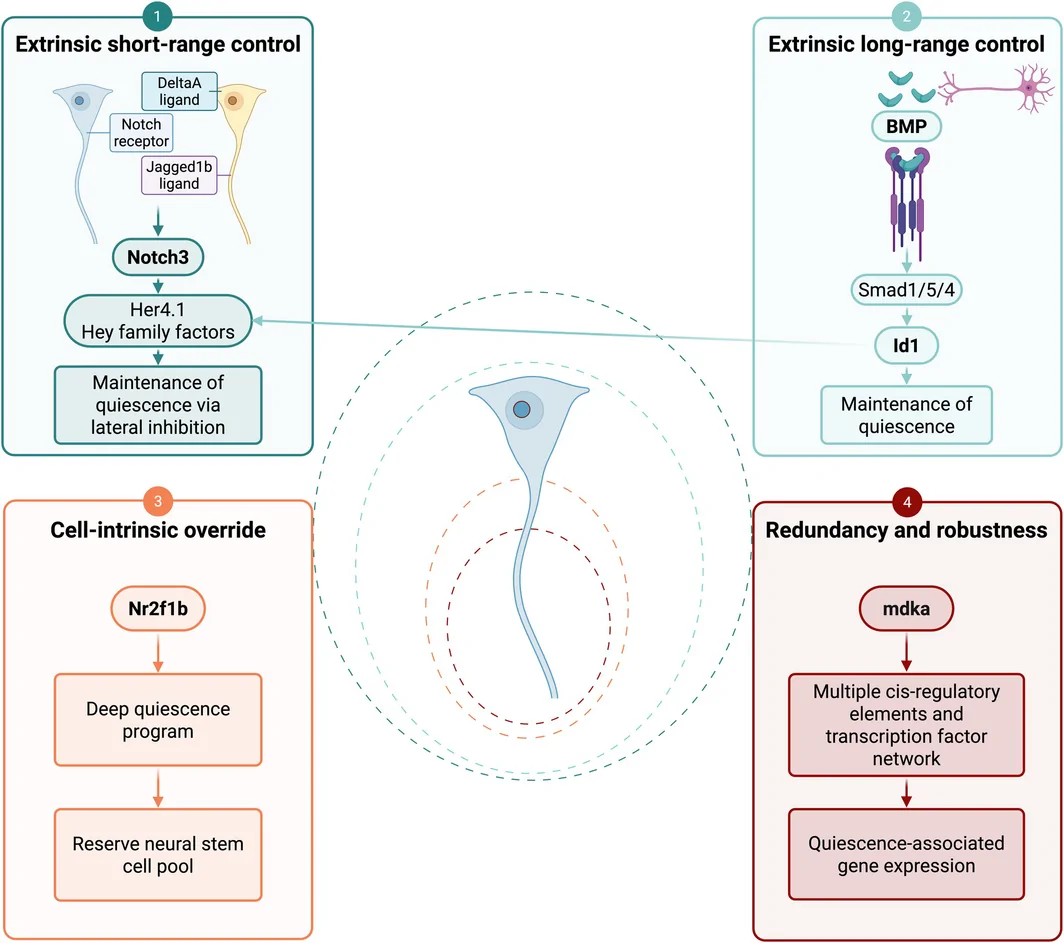
Proposed integration of the four regulatory inputs controlling RGC quiescence (hypothesis; see Section 6). (1) short‐range Notch3 signaling from neighboring Type 2 progenitors; (2) long‐range BMP signaling from distant neurons acting through Smad proteins to induce Id1; (3) cell‐intrinsic quiescence mediated by nuclear receptor Nr2f1b; and (4) Mdka, a secreted factor proposed, but not yet demonstrated by loss of function, to act as a pathway‐independent buffer. Her4.1 is shown as the proposed convergence node integrating the Notch3 and BMP/Id1 pathways (inputs 1 and 2). The rostral‐caudal axis of the pallium is indicated: Proliferation is highest rostrally, whereas Nr2f1b‐enriched, activation‐resistant RGCs are enriched caudally, close to the choroid plexus, a candidate posterior source of secreted modulators (Section 6.2). The arrangement into a hierarchy of inputs is the hypothesis developed in Section 6 and not an established fact. Created in BioRender. Hoareau (2026) https://BioRender.com/7tbt1y6.

We propose that this arrangement maps onto the quiescence continuum described in Section 3 (Figure 3). Primed quiescent RGCs would rely mainly on the two extrinsic inputs and on their convergence on Her4.1. Deeply quiescent RGCs would rely mainly on Nr2f1b with a possible contribution from *mdka*, whereas intermediate states would result from graded contributions of all four. Decoupling from extrinsic control has been demonstrated only for Notch and BMP inhibition, under the conditions described in Section 4.3. The implication of the proposal is that different subpopulations operate under different regulatory configurations, so that distinct interventions would be required to engage each of them.

### 6.2 A Spatial Dimension: Regionalization Along the Antero‐Posterior Axis

The framework so far treats the four inputs as if they were uniformly available across the pallial ventricular zone. They are not. Proliferation follows a rostral‐high to caudal‐low gradient, and the Nr2f1b‐enriched, activation‐resistant population lies along the opposite gradient (Morizet et al. 2025). *id1* is likewise not uniformly distributed, and the BMP ligands that drive it come from parenchymal neurons whose density varies regionally (Rodriguez Viales et al. 2015; Zhang et al. 2021). We therefore propose, as the spatial component of the hypothesis, that the cell‐intrinsic Nr2f1b input predominates caudally and the two extrinsic inputs rostrally, so that interactions between them should be detectable mainly in the caudal domain, where the relevant populations overlap.

A candidate physical basis already exists. The choroid plexus and tela choroidea occupy the posterior telencephalic region and express BMP agonists, an angiopoietin‐like protein capable of acting as a Notch agonist, and the Wnt inhibitor Dkk3b (Morizet et al. 2025). A posterior secretory source of this kind would provide a simple basis for graded, position‐dependent signaling, and it can be tested by localized manipulation of the choroid plexus. This also gives a concrete answer to how the state of one cell relates to that of its neighbors and of cells at a distance: contact‐dependent Notch couples a cell only to adjacent cells; diffusible BMP and choroid plexus‐derived factors couple it to its position in the tissue; and the cell‐intrinsic Nr2f1b program is insensitive to both. The mosaic of activation propensities across the ventricular zone would then follow from the superposition of a contact‐dependent, a graded, and a position‐independent input, rather than from cell‐to‐cell variability alone.

### 6.3 An Alternative Interpretation We Cannot Yet Exclude

Because deep quiescence and caudal position are correlated, an alternative to this account is that the continuum of quiescence depth is in substantial part a spatial gradient read out at the population level, cells differing less in an intrinsic property than in their position relative to the signal sources. The two interpretations make different predictions. If depth is primarily positional, a caudal Nr2f1b‐high RGC placed in a rostral environment should adopt rostral behavior; if depth is a cell‐autonomous state, it should not, at least not rapidly. We regard this as the most important single test of the framework, and the available data do not decide between the two.

### 6.4 Predictions and How They Could Be Tested

Because the framework proposed in this section is a hypothesis rather than an established mechanism, Table 4 sets out the predictions it generates together with the experiments that would confirm or refute them. We regard these predictions, rather than the framework itself, as the principal contribution of this synthesis.

**TABLE 4:** Predictions generated by the integrative framework proposed in Section 6.

Claim of the framework	Testable prediction	Experimental test
The four pathways act within the same cell, with their relative weight shifting along the continuum	Individual RGCs co‐express the four readouts in graded combinations; deeply quiescent cells lose dependence on Notch/BMP output	Single‐cell and spatial multi‐omics along the ventricular zone; conditional manipulation of each input in cells of defined state
BMP‐Id1 stabilizes Notch output through Her4.1 (convergence model)	BMP inhibition lowers and destabilizes *her4.1* dynamics even where Notch input is intact	Live *her4.1*‐reporter imaging with and without DMH1; Id1 gain‐ and loss‐of‐function; direct test of Her4.1 autorepression relief
Neighbor and distance effects: local BMP availability sets a mosaic of activation thresholds	RGCs distal from BMP‐secreting neurons show lower Her4.1 and higher activation propensity	Spatial transcriptomics with distance‐to‐neuron mapping; localized manipulation of BMP source
Commitment to the Nr2f1b state depends on activation history, age or metabolic state	Cells progressively acquire Nr2f1b‐dependent maintenance as a function of prior activation or age	Clonal lineage tracing of activation history combined with an Nr2f1b reporter across ages
Midkine‐a acts as a pro‐quiescence buffer	Loss of *mdka* causes premature RGC activation	*mdka* mutant or conditional knockdown with proliferation and activation readouts
The weighting of the four inputs is regionally biased along the antero‐posterior axis	Nr2f1b‐dependent maintenance predominates caudally and extrinsic control rostrally; interactions between them are detectable principally in the caudal pallium	Region‐resolved perturbation of Notch, BMP and Nr2f1b in rostral versus caudal pallium; localized manipulation of the choroid plexus as a candidate posterior signaling source
Quiescence depth is a cell‐autonomous state rather than a read‐out of position (Section 6.3)	A caudal, Nr2f1b‐high RGC retains its low activation propensity when placed in a rostral environment	Transplantation or region‐specific clonal tracking combined with an Nr2f1b reporter

## 7 Conservation and Implications for Vertebrate Neural Stem Cell Biology and Regeneration

The regulatory logic described in the adult zebrafish telencephalon is not an idiosyncratic feature of teleost biology. Its principal components are broadly conserved across vertebrates and understanding how they cooperate in zebrafish offers direct insights into the regulation and dysfunction of mammalian neural stem cell niches. The comparison below is restricted to the conservation of the regulatory mechanisms and to what is securely established about regenerative outcome in mammals.

### 7.1 Conserved Molecular Parallels With Mammalian Neurogenic Niches

In the adult mouse brain, neural stem cells in the SVZ and dentate gyrus are similarly regulated by both Notch and BMP signaling (Ables et al. 2010; Basak et al. 2012; Bond et al. 2014; Choe et al. 2015; Diotel et al. 2020; Kawai et al. 2017; Lim et al. 2000; Mira et al. 2010; Urban et al. 2019; Urban and Guillemot 2014). In the SVZ, Notch signaling maintains quiescence through *Hes1* and *Hes5*, whose sustained expression suppresses proneural gene activity and prevents stem cell activation (Sueda et al. 2019; Sueda and Kageyama 2020). BMP signaling induces Id proteins, which are directly implicated in preserving stem cell dormancy: Id1 marks and maintains B1‐type stem cells in the SVZ (Nam and Benezra 2009), and Id4, acting downstream of Notch2 signaling in the dentate gyrus, blocks cell‐cycle entry in quiescent radial glia (Zhang et al. 2019). The parallel to the zebrafish Id1 mechanism is striking. Functional evidence reinforces this parallel: in the adult mouse, Smad4‐mediated BMP signaling in stem cells is required to initiate the neurogenic lineage, and its loss impairs neurogenesis (Colak et al. 2008). The Her4.1 convergence model proposed here, whereby Id proteins stabilize Notch pathway output by attenuating Hes/Her autorepression, may therefore represent a conserved mechanism operating in the mammalian brain as well (Bai et al. 2007; Boareto et al. 2017). Direct evidence for BMP‐to‐Notch pathway coupling in neural stem cells remains to be established, although the broader principle is supported by converging evidence from other stem cell contexts. In osteoblasts, BMP9 directly induces *Hes1* transcription through SMAD signaling independently of canonical Notch receptor activation (Seong et al. 2021). More recently, genome‐wide cleavage under targets and tagmentation (CUT&Tag) profiling in adult muscle stem cells demonstrated that BMP4 and BMP6 rapidly induced expression of *Hes1*, *Hey1*, and other Notch pathway components, with direct pSMAD1/5/9 and SMAD4 occupancy detected at their regulatory elements (Lange et al. 2026). Together, these findings from multiple adult stem cell niches raise the possibility that BMP‐driven reinforcement of Notch pathway output is a general principle of adult stem cell regulation that may well extend to the neural context.

The cell‐intrinsic Nr2f1b mechanism is similarly conserved. NR2F1 (COUP‐TF1), the mammalian orthologue of zebrafish Nr2f1b, promotes long‐term neural stem cell dormancy in the mouse, and its activity has been linked to the establishment of a reserve stem cell pool in the adult SVZ (Tocco et al. 2021). Deeply quiescent NR2F1‐positive cells in the mouse brain share several transcriptional features with their zebrafish counterparts, including enrichment for Fabp7/Blbp and a low‐Her/Hes signature, further supporting the conservation of this regulatory node across vertebrates (Morizet et al. 2025).

### 7.2 Regenerative Outcome in Zebrafish and Mammals

One of the most compelling reasons to study neural stem cell quiescence in zebrafish is the stark difference in regenerative outcome between teleosts and mammals after brain injury. Following telencephalic injuries, zebrafish efficiently reactivate quiescent RGCs, mount a robust neurogenic response, and achieve substantial structural and functional repair (Baumgart et al. 2012; Kizil, Kyritsis, et al. 2012; Kroehne et al. 2011; Kyritsis et al. 2012; März et al. 2011). In contrast, adult mammals achieve only limited functional repair. The resident NSC pool does decline with aging (Encinas et al. 2011; Kempermann et al. 2018; Obernier and Alvarez‐Buylla 2019; Urban et al. 2019), and the post‐injury inflammatory environment is prolonged and poorly resolved, with sustained microglial activation, reactive astrogliosis and glial scar formation (Chen et al. 2024; Clain et al. 2025; Diotel et al. 2020). Critically, however, the deficit is not a failure to mount neurogenesis. Following injury such as stroke, neuroblast production from adult SVZ stem cells is not reduced but continues without decline for at least 4 months (Thored et al. 2006). The limiting step is instead the survival and circuit integration of the newborn neurons: a fraction mature and persist, but many die by caspase‐mediated apoptosis (Thored et al. 2006), in part because they retain the subtype identity of their niche of origin (e.g., olfactory‐bulb interneuron identity from the SVZ) rather than that of the damaged tissue. The dentate gyrus behaves differently: neurogenesis can eventually decline in long‐standing temporal lobe epilepsy, but this appears to follow prolonged sclerosis, and the neurons principally lost (CA1/CA3) are in any case not those generated by adult SGZ stem cells. In zebrafish, on the other hand, the post‐injury inflammatory response is transient and tightly controlled: microglial activation resolves rapidly without formation of a glial scar or deposition of inhibitory extracellular matrix, generating a permissive environment for RGC reactivation and neurogenesis (Chen et al. 2024).

The framework proposed in Section 6 suggests one way of understanding this difference. The following paragraph is therefore interpretation rather than established fact. In zebrafish, the existence of a deeply quiescent Nr2f1b‐positive reserve, protected from casual activation by both cell‐intrinsic mechanisms and pathway‐redundant *mdka* buffering, means that even after extensive activation of the primed and intermediate populations, a pool of stem cells remains that can sustain long‐term regeneration without depleting the compartment. The regulation of this reserve is tight enough to prevent accidental activation yet reversible enough to allow recruitment under the strong signals associated with injury. In the mammalian brain, evidence for a directly equivalent NR2F1‐positive reserve population is emerging (Tocco et al. 2021). However, its reactivation under physiological conditions appears far more restricted, possibly because additional layers of inhibition imposed by the gliogenic niche environment, inhibitory extracellular matrix components, and progressive reduction in pro‐neurogenic growth factor availability further dampen reactivation signals that would be sufficient in zebrafish. It should be emphasized, however, that in the mammalian brain, the principal barrier identified to date lies downstream of activation: newly generated neurons are produced in substantial numbers after injury but largely fail to survive and integrate, so that strategies aimed solely at enhancing stem cell recruitment are unlikely to be sufficient on their own (Encinas et al. 2011; Obernier and Alvarez‐Buylla 2019). From this perspective, the regenerative advantage of the zebrafish system appears to extend beyond the mere abundance of stem cells. Instead, it would depend on a quiescence program that preserves a long‐term stem cell reserve capable of regulated reactivation.

### 7.3 Implications for Neural Repair Strategies

These insights carry concrete implications for the design of neural repair strategies. Rather than targeting a single pathway to broadly reactivate neural stem cells, an approach likely to exhaust the primed and intermediate pools without engaging the deeply quiescent reserve, future interventions may need to be stratified according to the quiescence state of the target population. For the primed compartment, modulation of Notch or BMP signaling may be sufficient. For the deeply quiescent reserve, transiently reducing Nr2f1b activity or its downstream effectors may be necessary to render these cells responsive to activation signals; however, any approach targeting Nr2f1b would require cell‐type‐specific delivery, since NR2F1 is expressed broadly beyond neural stem cells and systemic manipulation risks unintended effects in other NR2F1‐expressing tissues. The *mdka* pathway presents a particularly interesting complementary target: because *mdka* expression depends on multiple converging inputs, disrupting specific regulatory elements rather than the signaling pathways themselves may offer a more precise means of modulating quiescence in this population. Identifying the receptor and downstream pathway through which *mdka* acts will be a critical step toward this goal.

More broadly, these findings argue for a precision approach to neural stem cell biology in which therapeutic strategies are designed with explicit awareness of the quiescence state being targeted, the regulatory configuration that state operates under, and the combination of signals required to achieve controlled activation without pool exhaustion.

## 8 Conclusion and Perspectives

The adult zebrafish telencephalon has emerged as a uniquely tractable system for dissecting the molecular logic of neural stem cell quiescence. Rather than a simple on/off switch, quiescence exists along a continuum of molecularly distinct states, controlled by the four mechanisms reviewed in Section 4 and summarized in Table 3.

The central proposal of this review is that the quiescence spectrum reflects a transition between two modes of maintenance: primed cells environmentally controlled, deeply quiescent cells cell‐autonomously controlled. As set out in Section 7.2, however, the difference in regenerative outcome between zebrafish and mammals is not reducible to stem cell biology alone, since the resolution of the post‐injury inflammatory environment differs markedly between them. How quiescence regulation and inflammatory resolution are coordinated, and whether manipulating one can compensate for deficits in the other, is an important frontier for regenerative neuroscience. We emphasize that the limited regenerative outcome in mammals is not attributable to a failure of injury‐induced neurogenesis, which can persist for months, but principally to the poor survival and circuit integration of the neurons produced.

Several important questions define the frontier of the field. How is the transition from environmentally regulated to cell‐autonomously regulated quiescence triggered, and what determines when a cell commits to Nr2f1b‐dependent maintenance? Does Nr2f1b expression respond to cumulative activation history, aging signals, or metabolic state? What is the receptor for Mdka, and through what downstream pathway does it sustain quiescence‐associated gene expression? How are the four mechanisms remodeled during aging, injury, and disease, and do the proportions of cells in each quiescence state shift in ways that explain the decline of regenerative capacity in older animals? And how does the inflammatory microenvironment interact with these mechanisms: does early resolution of inflammation in zebrafish directly lower the activation threshold of primed RGCs, or does it operate through independent niche signals?

Addressing these questions will require the integration of single‐cell multi‐omics with spatial transcriptomics, clonal lineage tracing, and targeted perturbation studies at cellular resolution. The comparative analysis of quiescence regulation across vertebrate species, exploiting the mechanistic conservation between zebrafish and mammals highlighted in this review, will be equally important. Ultimately, understanding how quiescence is actively generated, maintained, and reversibly regulated in a highly regenerative vertebrate, and how this regulation is embedded within a broader pro‐regenerative inflammatory context may yield the mechanistic insights needed to unlock latent regenerative potential in the mammalian brain and develop rational strategies for neural repair.

## Author Contributions

M.H.: literature review, manuscript drafting. N.D.: conceptual input, critical revision. S.R.: conceptualization, supervision, funding acquisition, writing – original draft, critical revision. All authors approved the final manuscript.

## Funding

This work was supported by the Marie Skłodowska‐Curie Doctoral Network DANIO‐ReCODE (Horizon Europe grant agreement No. 101169349).

## Conflicts of Interest

The authors declare no conflicts of interest.

## Data Availability

Data sharing not applicable to this article as no datasets were generated or analysed during the current study.
